# Tertiary lymphoid structures in cancer: maturation and induction

**DOI:** 10.3389/fimmu.2024.1369626

**Published:** 2024-04-16

**Authors:** Yulu Chen, Yuhao Wu, Guorong Yan, Guolong Zhang

**Affiliations:** ^1^ Department of Phototherapy, Shanghai Skin Disease Hospital, School of Medicine, Tongji University, Shanghai, China; ^2^ Skin Cancer Center, Shanghai Skin Disease Hospital, School of Medicine, Tongji University, Shanghai, China; ^3^ Institute of Photomedicine, School of Medicine, Tongji University, Shanghai, China

**Keywords:** tertiary lymphoid structure, immunotherapy, cancer treatment, tumor microenvironment, maturity

## Abstract

Tertiary lymphoid structure (TLS) is an ectopic lymphocyte aggregate formed in peripheral non-lymphoid tissues, including inflamed or cancerous tissue. Tumor-associated TLS serves as a prominent center of antigen presentation and adaptive immune activation within the periphery, which has exhibited positive prognostic value in various cancers. In recent years, the concept of maturity regarding TLS has been proposed and mature TLS, characterized by well-developed germinal centers, exhibits a more potent tumor-suppressive capacity with stronger significance. Meanwhile, more and more evidence showed that TLS can be induced by therapeutic interventions during cancer treatments. Thus, the evaluation of TLS maturity and the therapeutic interventions that induce its formation are critical issues in current TLS research. In this review, we aim to provide a comprehensive summary of the existing classifications for TLS maturity and therapeutic strategies capable of inducing its formation in tumors.

## 1 Introduction

Tumors represent a complex ecosystem, and there may be substantial variations in the composition and functional status of the tumor microenvironment (TME) across different tumor categories, intrinsic characteristics, stages, and patient conditions. The TME is a highly organized system comprising diverse immune cells, tumor-associated fibroblasts, endothelial cells, and extracellular matrix, with these constituents exhibiting variability based on tissue types and co-evolving with tumor advancement. Tertiary lymphoid structures (TLS), previously termed “tertiary/ectopic lymphoid organ/structure” and “inducible lymphoid organ,” are organized aggregates of immune cells within the TME, characterized by a central B cell zone encircled by a rich T cell zone, akin to secondary lymphoid structures (SLO) (1). The composition of TLS is diverse. The primary subset of T cells within TLS is CD4^+^ T follicular helper (Tfh) cells, accompanied by CD8^+^ cytotoxic T cells, CD4^+^ T helper 1 (Th1) cells, and regulatory T cells (Tregs). CD21^+^ follicular dendritic cells (FDC) may also be found in TLS, playing a crucial role in memory B cell selection within the germinal center. CD83+ mature DC expressing dendritic cell lysosome-associated membrane protein (DC-LAMP^+^) are predominantly situated in the T cell zone. CD68^+^ macrophages are sporadically present in some TLS, contributing to the clearance of apoptotic cells (2). Furthermore, numerous stromal cells resembling follicular reticular cells in SLO help anchor TLS in areas of chronic inflammation (3, 4). The peripheral lymph node address protein (PNAd)-positive high endothelial venules (HEV) create specialized vasculature for TLS, facilitating lymphocyte recruitment. Although TLS shares anatomical structure and function with SLO, there are notable differences between them. TLS is directly exposed to the TME due to the absence of fibrous capsules. This allows antigen-presenting cells and lymphocytes to efficiently and rapidly recognize antigens, avoiding the need to travel between tumor tissues and SLO (5). SLO is programmed and orderly formed during embryogenesis, whereas TLS is distributed locally at sites of inflammation and not present under healthy conditions (6). Unlike SLO characterized by well-defined T and B compartments (7), TLS exhibits morphological diversity, ranging from preliminary mixtures of T and B cell clusters to elaborate T and B compartment formations including germinal centers (GC).

Many clinical studies have found that TLS is associated with a good prognosis of cancer patients, and it can also be used as a predictor of the efficacy of cancer treatment, especially for immunotherapy (8, 9). TLS has immature and mature state. In immature TLS, there is scant evidence of inducing effective immune responses, primarily linked to T cell exhaustion, inflammation, and/or an immunosuppressed TME (10, 11). B cells within immature TLS are scarce and typically produce low-affinity antibodies. In contrast, mature TLS is characterized by the presence of GC containing BCL6^+^ B cells, promoting the selective activation and expansion of B-cell clones. This process facilitates antibody class switching and somatic hypermutation. The activated B cells in mature TLS can differentiate into plasma cells that secrete antibodies of high affinity, demonstrating heightened immune activity (12). The biological significance of B cell-induced immune responses in promoting or inhibiting cancer development is unclear. Some studies have found that when B cells in the context of TLS, they are associated with better prognosis and are the core of the anti-tumor effects of TLS (13). Consequently, there is a growing emphasis on enhancing the anti-tumor immune response by inducing TLS formation. Notably, various cancer treatments have been identified as capable of triggering TLS formation. These include immunotherapy, chemoradiotherapy, cytokines, agonists, inhibitors, combination therapies, as well as potential treatments like microbial transplantation and exogenous vesicle injection. For example, vaccination with the human papillomavirus (HPV) oncoprotein vaccine and the pancreatic cancer vaccine induce the formation of TLS (14, 15). Radiotherapy combined with targeted Tregs therapy improve the immunosuppressive microenvironment of glioma and induce the formation of meningeal TLS (16). This review provides a comprehensive overview of TLS formation, detection, maturity, and induction through various cancer treatments, highlighting the clinical significance of TLS and laying the groundwork for future anti-cancer therapies that target TLS formation.

## 2 Formation of TLS

**Figure 1:**
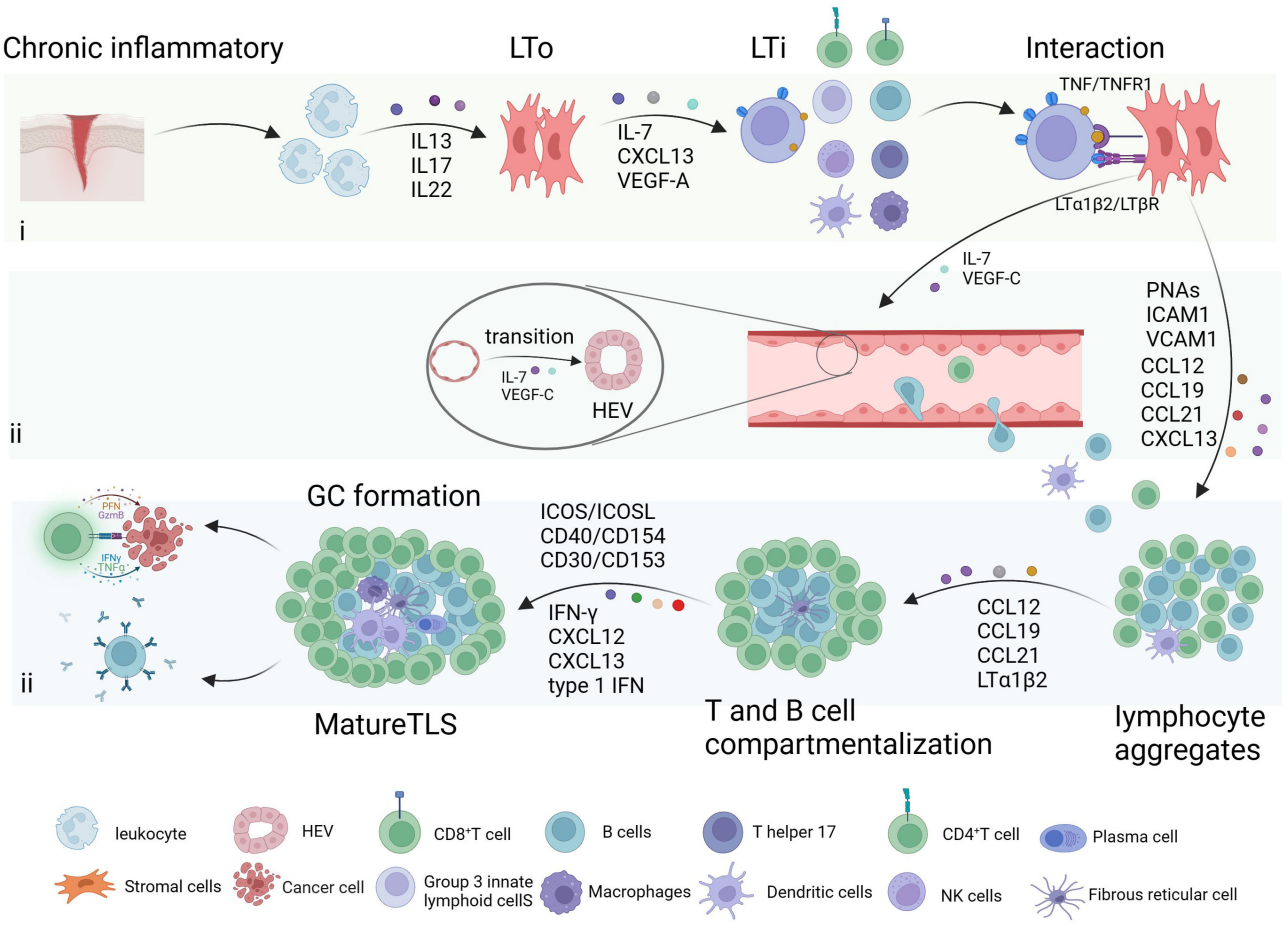
Formation of tertiary lymphoid structure. The formation of tertiary lymphoid structure (TLS) is a response to inflammatory stimulation mediated by different cytokines, and it can be divided into three steps: (i) interaction of lymphoid tissue inducer cells (LTi) and lymphoid tissue organizers (LTo); (ii) high endothelial venules (HEV) formation and immune cells recruitment; (iii) T/B compartmentalization and germinal centers (GC) formation. Created with BioRender.com.

The formation of TLS is a response to chronic inflammatory or tissue injury irritation and can be divided into three steps: (i) interaction of lymphoid tissue inducer cells (LTi) and lymphoid tissue organizers (LTo); (ii) HEV formation and immune cells recruitment; (iii) T/B compartmentalization and GC formation (**Figure 1**).

Leukocytes attract by chronic inflammation or tissue damage release interleukin 13 (IL-13), IL-17, and IL-22, induce stromal cells activated adjacent to inflammation (4). Stimulated stromal cells and lymphocytes then secrete CXC-chemokine ligand 13 (CXCL13), IL-7, and vascular endothelial growth factor (VEGF)-A, which induce LTi cell recruitment (17). LTi cells or other immune cells, including innate lymphoid cells 3, IL-17-producing T helper (Th17) cells, effector CD8^+^ T cells, B cells, and M1 macrophages, have the potential to initiate TLS formation by interacting with lymphotoxin beta receptor (LTβR) and tumor necrosis factor receptor 1 (TNFR1) on LTo cells/stromal cells through LTα1β2 and Tumor Necrosis Factor (TNF) (18). This interaction promotes LTo cells to produce adhesion molecules such as VEGF-C, vascular cell adhesion molecule 1, intercellular adhesion molecule 1, and peptide nucleic acids (PNAs), as well as chemokines such as IL-7, CC-chemokine ligand 12 (CCL12), CCL19, CCL21, and CXCL13. These factors play a key role in the formation of HEV and the recruitment of immune cells. For example, VEGF-C and IL-7 promote the transition of the endothelial vascular system from flat to high endothelium (19). T and B cell compartmentalization occurs as a result of specific recruitment by cytokines like CXCL12, CXCL13, CCL19, CCL21, and LTα1β2 (20). In the GC, the segregation into dark and light zones is guided by CXCL12 and CXCL13 (21). IFN-γ triggers B cell activation by upregulating BAFF, APRIL, and IL-6 (22). CXCL13 induces B cells to differentiate into antibody-producing cells, while IFN1 recruits CXCR5^+^ B cells and promotes the development of Tfh cells (23). Subsequently, Tfh cells interact with B cells through ICOSL/ICOS, CD40/CD154, CD30/CD153 interactions, driving isotype switching, affinity maturation, and B‐cell differentiation (21, 24).

## 3 Detection of TLS

It is important to find effective and reliable predictors for early detection of TLS. The detection of TLS predominantly relies on pathological diagnosis, including Hematoxylin and Eosin (HE) staining, Immunohistochemistry (IHC), and Immunofluorescence (IF) (25). The detection of TLS predominantly relies on pathological diagnosis, including Hematoxylin and Eosin (HE) staining, Immunohistochemistry (IHC), and Immunofluorescence (IF) (25). Immunostaining stands as the foremost method for identifying TLS. HE staining is widely accepted for the detection of TLS due to its simplicity and affordability, but it is easy to cause misjudgment owing to its limited detail or insufficient information. IHC and IF techniques are employed to qualitatively identify TLS by staining specific markers expressed by immune cells within TLS (26). These techniques also facilitate the analysis of cellular composition and maturation of TLS. Based on these techniques, multiple immunofluorescence has been developed to further analyze the spatial structure of TLS and enable simultaneous detection and visualization of multiple target proteins in a single sample, providing the possibility to observe spatial conformation and cellular interactions (27). Worth noting, false negative results can arise from the failure to collect tumor tissue or when dealing with atypical samples.

The gene signature associated with TLS provides more objective evidence compared to morphological observation for the TLS detection. The 12-chemokines signature (CCL2, CCL3, CCL4, CCL5, CCL8, CCL18, CCL19, CCL21, CXCL9, CXCL10, CXCL11, and CXCL13) contains genes encoding myeloid, T cell attractants, and B cell attractants, and has been utilized to detect TLS in human colorectal cancer, melanoma, hepatocellular carcinoma, and breast cancer. This signature is the most widely used biomarker and is employed to detect all types of TLS (28). CXCL13 is recognized as a straightforward transcriptomic marker (29, 30). Spatial transcriptomics found a 29-gene TLS imprint signature enriched with immunoglobulin genes, plasma cell genes, and T cell markers. It characterizes mature TLS, where B cell maturation towards plasma cell takes place (31). Additionally, other signatures including a 9-gene signature, an 8-gene Tfh signature (32), a Th1/B cell signature (33), and a plasma cell signature-TNFRsf17 (3) can also be utilized for TLS detection (**Table 1**).

**Table 1:** The gene signature of tertiary lymphoid structure.

Signature genes	Genes	Method	Refs
12- chemokines signature	CCL2, CCL3, CCL4, CCL5, CCL8, CCL18, CCL19, CCL21, CXCL9, CXCL10, CXCL11, CXCL13	mRNA microarray analysis	(28)
9-chemokines signature	PTGDS, RBP5, EIF1AY, CETP, SKAP1, LAT, CCR6, CD1D, CD79B	RNA-seq analysis	(34)
Tfh cell signature	CXCL13, CD200, FBLN7, ICOS, SGPP2, SH2D1A, TIGIT, PDCD1	qRT-PCR	(32)
Th1/B cell signature	CD4, CCR5, CXCR3, CSF2, IGSF6, IL-2RA, CD38, CD40, CD5, MS4A1, SDC1, GFI1, IL-1R1, IL-1R2, IL-10, CCL20, TRAF6, STAT5A	mRNA microarray	(33)
Plasma cell signature	TNFRSF17, IGJ	NanoString gene expression analysis	(3)
CXCL13	CXCL13	RNA-seq analysis	(29, 30)
TLS imprint signature	IGHA1, IGHG1, IGHG2, IGHG3, IGHG4, IGHGP, IGHM, IGKC, IGLC1, IGLC2, IGLC3, JCHAIN, CD79A, FCRL5, MZB1, SSR4, XBP1, TRBC2, IL-7R, CXCL12, LUM, C1QA, C7, CD52, APOE, PTLP, PTGDS, PIM2, DERL3	spatial transcriptomic analysis	(31)

CCL, CC- chemokine ligand; CCR5, CC- chemokine receptor 5; CSF2, colony- stimulating factor 2; CXCL, CXC- chemokine ligand; CXCR3, CXC- chemokine receptor 3; FBLN7, fibulin 7; ICOS, inducible T cell co- stimulator; IGSF6, immunoglobulin superfamily member 6; IL, interleukin; IL-1R, interleukin-1 receptor; SDC1, syndecan 1; SGPP2, sphingosine-1-phosphate phosphatase 2; SH2D1A, SH2 domain- containing protein 1 A; STAT5A, signal transducer and activator of transcription 5A; TFH cell, T follicular helper cell; TH cell, T helper cell; TIGIT, T cell immunoreceptor with Ig and ITIM domains; TLS, tertiary lymphoid structure; TNF, tumour necrosis factor; TNFRSF, tumour necrosis factor receptor superfamily member; TRAF6, tumour necrosis factor- receptor-associated factor 6.

Spatial transcriptomics and spatial proteomics offer valuable insights into spatially resolved gene expression and protein localization within complex biological systems, enabling a deeper understanding of cellular heterogeneity, tissue architecture, disease mechanisms, and potential therapeutic interventions (35). Continued advancements in these technologies paved the way for a deeper understanding of TLS, and hold great promise for accelerating biomedical research and precision medicine initiatives.

Nowadays, the identification and classification of TLS rely on manual detection, which is an empirical and potentially inaccurate process that is also time-consuming. Relying on artificial intelligence technology to detect TLS instead of manual detection has attracted great attention. In particular, computer learning programs that rely on traditional HE staining images have been developed and have demonstrated the ability to automatically detect, count, and classify TLS in gastrointestinal cancers and lung adenocarcinomas with high accuracy (36, 37).

## 4 Clinical value of TLS

Tumors-TME is classified into three types: infiltrated, excluded and desert phenotypes. The desert subtype is characterized by a scarcity of CD8^+^ T cells. The key distinction between the infiltrated and excluded subtypes lies in the spatial distribution of CD8^+^ T cells. In the infiltrated subtype, CD8^+^ T cells are located within the tumor epithelium, whereas in the excluded subtype, they are located within the tumor stroma (38). TLS tends to occur in dispersed and structured TME. Previous studies on the association between TLS and tumor prognosis have produced conflicting results, with tumor-promoting or tumor-inhibiting effects (39–41). Nevertheless, an important consistent finding emerged when considering the maturity of TLS: mature TLS was found to be associated with better prognosis in various solid tumors (42).

Studies that solely examined the spatial distribution of TLS within tumors, without taking into account their maturity, have yielded different findings. For example, in non-metastatic colorectal cancer, high-density peritumoral TLS was positively associated with improved recurrence-free survival and overall survival (OS), and was an independent and favorable prognostic factor for patients, while there was no correlation in intratumoral TLS (43). In two independent cohorts of hepatocellular carcinoma patients, peritumoral TLS demonstrated a strong association with a favorable prognosis. Tumors exhibiting high-density peritumoral TLS exhibited significantly higher percentages of CD3^+^, CD8^+^ and CD20^+^ cells, while showing decreased percentages of forkhead box P3^+^, CD68^+^ and programmed cell death 1 (PD1^+^) cells in the TME (44). However, studies of other cancers such as intrahepatic cholangiocarcinoma, colorectal cancer, breast cancer, hepatocellular carcinoma, and cholangiocarcinoma have shown that intratumoral TLS was positively associated with better clinical outcomes, while peritumoral TLS was linked to a worse prognosis (45–48). These findings contradict the earlier conclusions. With an increased understanding of TLS, attention has been directed toward the maturity of TLS, revealing that mature TLS is linked to better cancer prognosis (49).

In a large retrospective analysis of 540 patients with different types of cancer patients treated with PD1/PD-L1 blockade, it was observed that the presence of mature TLS was significantly correlated with improved objective response rate, progression-free survival, and OS. This association was found to be independent of PD-L1 expression levels and the number of CD8^+^ T cells (50). In Epstein-Barr virus-associated gastric cancer, the presence of intratumoral mature TLS serves as an independent predictor of OS and is associated with a favorable response to neoadjuvant chemotherapy and anti-PD-1 therapy (51). Besides, the maturity of TLS can be affected by tumor type, location, primary or metastatic status, etc. For example, TLS in primary melanoma is in an immature state lacking GC and mostly located within the tumor. On the contrary, TLS in metastatic melanoma is primarily located at the tumor periphery with visible GC (52). In urothelial cancer, TLS located in the superficial layer of the urethral mucosa appear as a structureless lymphocyte aggregates, whereas the deep layer TLS exhibit a more mature state of follicular structure (53).

In conclusion, prior research has reported contradictory effects of TLS in various types of tumors, possibly due to the oversight of TLS maturity. Recent studies have consistently indicated that mature TLS is correlated with improved prognosis in cancer patients. This suggests that future investigations pertaining to TLS should prioritize the assessment of TLS maturity.

## 5 Maturity of TLS

**Figure 2:**
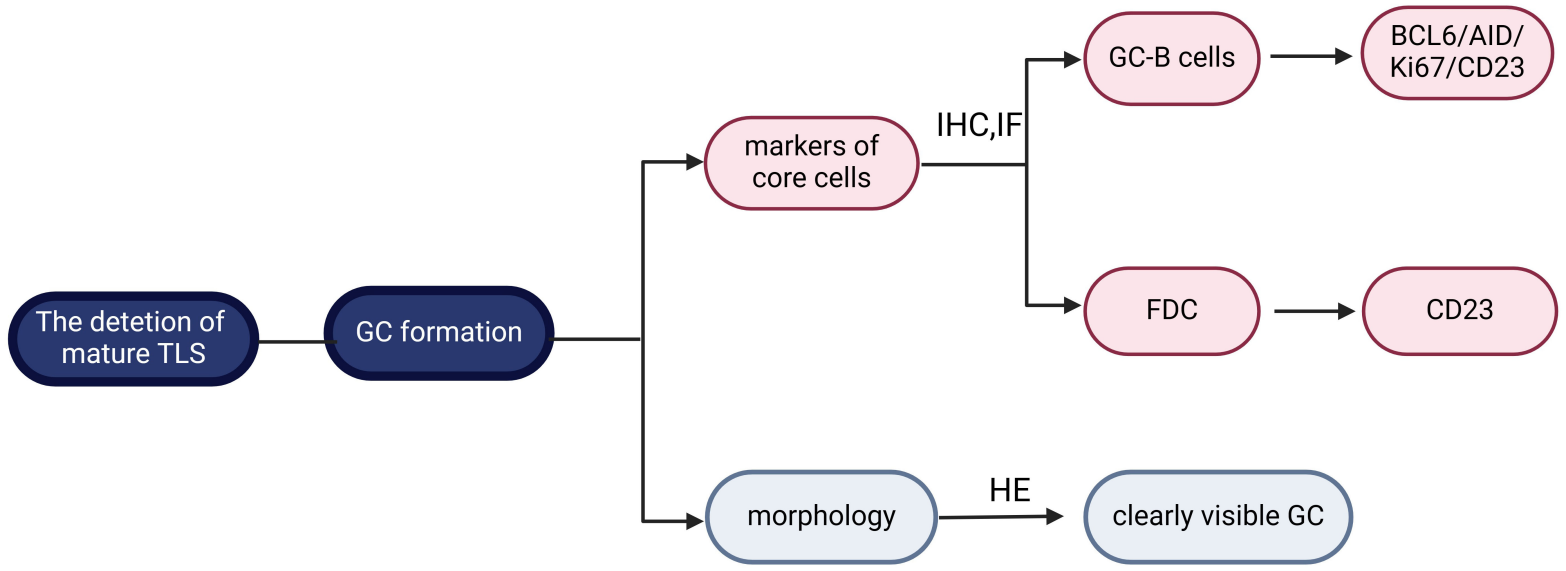
Maturity classification of tertiary lymphoid structure. The defining criterion for mature tertiary lymphoid structure (TLS) is germinal centers (GC) formation. It can be further classified according to morphology or follicular dendritic cells (FDC)/GC-B cell markers. Created with BioRender.com.

Mature TLS exhibits cellular aggregates characterized by follicular structures housing GC, thereby demonstrating enhanced immune functionality. HEV represents specialized vessels within mature TLS, facilitating the transport of lymphocytes. Additionally, the induction of FDC within mature TLS is mediated by LT and TNF, promoting the development of B cell-rich regions (6). Currently, the criterion for TLS maturity is the formation of GC, which is identified by specific morphological and molecular characteristics. GC is a dynamic region with a network of CD21^+^/CD23^+^ FDC and B-cell lymphoma 6 protein (BCL6)^+^ CD20^+^ B cells, primarily involved in B cell activation, proliferation, and differentiation during immune response (27, 30, 31). If the GC structure was clearly visible under HE, it was considered to be mature TLS (54); otherwise, it required to further identification of CD21^+^/CD23^+^ FDC and BCL6^+^ CD20^+^ B cells in GC by IHC and IF (**Figure 2**).

Initially, CD21 positivity on FDC was thought to indicate GC formation (51, 55, 56). However, subsequent studies have shown decreased CD21 expression on FDC and increased CD23 expression with TLS maturation (12). Based on this, TLS has been divided into three stages of maturity: i) early TLS, characterized by dense lymphocytic clusters devoid of FDC and GC, and negative expression of CD21 and CD23; ii) primary follicle-like TLS, which consists of dense lymphocytic aggregates with a CD21^+^ FDC network but negative CD23 expression; iii) secondary follicle-like TLS, denoting the mature stage of TLS with active GC and CD21^+^CD23^+^ FDC (57). This classification reflects the maturation process of TLS, with only secondary follicle-like TLS considered mature due to the presence of GC. Therefore, some researchers have simplified the classification into “mature” and “immature” states based on CD23 positivity observed through IHC/IF (58). Besides, CD23 staining alone was more sensitive than CD20/CD23 dual staining for TLS detection (59).

Other researchers have defined the maturation stage of TLS by detecting markers linked to selective expansion and affinity maturation on B cells in GC, such as BCL6, Activation-induced cytidine deaminase, and Ki67 (8, 60). For example, Meylan et al. defined mature TLS as the presence of CD23^+^ FDC, BCL6^+^ GC-B cells, and PNAd^+^ HEV, whereas immature TLS had only CD20^+^ B cells, none of the above mentioned (31). Additionally, TLS maturity was assessed based on the abundance of DC-Lamp^+^ cells. TLS with a high density of DC-Lamp^+^ cells (≥1.5 cells/0.04 mm2) are classified as mature TLS, while TLS with a low density of DC-Lamp^+^ cells (<1.5 cells/0.04 mm2) are considered immature TLS (61). In summary, when the GC structure cannot be observed under HE, further evaluation of the critical cells involved in GC, such as CD21^+^/CD23^+^ FDC and BCL6^+^ CD20^+^ B cells, is necessary using IHC and IF techniques. Nevertheless, it remains unclear which of these cell types is more sensitive and specific in representing GC, requiring further investigation (**Table 2**).

**Table 2:** Maturity classification and markers of tertiary lymphoid structure.

Representative marker of GC	Tumor types	Auxiliary markers	Prognostic factors.	Species	Refs
HE	ESCC	N.A.	Presence	Human	(54)
	OSCC	IHC: CD20, Ki67, CD21, CD4, LAMP3, CD8	Maturity	Human	(62)
CD21	EBVaGC	IF: CD20, CD21, CD4, CD8, FOXP3	Location and maturity	Human	(51)
	HGSC	IHC: CD20, CD4, CD8, CD21, CXCL13	Presence	Human	(55)
	cSCC	IHC: CD20, CD3, CD8, MECA79, CD21	Presence	Human	(56)
CD23	Colorectal cancer	IF: CD3, CD20, CD21, CD23, CCL21, CXCL13	Maturity and density	Human	(57)
	LSCC	IHC: CD3, CD20, CD21, CD23, BCL2, BCL6 IF: CD3, CD20, CD23, CD21, PNAd, DC-LAMP, CXCL13	Density	Human and mice	(63)
	LSCC	IHC: CD20, CD21, CD23	Maturity	Human	(64)
	ESCC	IF: CD1c, CD23, CD4, CD8, CD19, CD21, CD138; IHC: CD21, CD23	Density	Human	(65)
	CCA	IHC: CD23, CD21, CD3, MECA-79, CD20, CD68, CD56	Location	Human	(48)
	Digestive tract cancer	IF: CD20, CD21, CD23, CD4, CD8, CD3, DC-LAMP, IgD, CXCR5, CD38, Podoplanin, AID, Bcl6, CD68, CD138, CD31, CD34, Ki67	N.A.	Human	(66)
	CMM	IF: CD20, CD21, CD23, CD8, Ki67, PNAd	Presence	Human	(67)
	Carcinomas and sarcomas	IHC: CD20, CD23	N.A.	Human	(59)
	UC	IF: PNAd, CD3, CD21, CD20, CD23, DC-LAMP	Presence	Human	(68)
	Solid tumors	IF: CD20, CD23, CD21, CD4, CD8; IHC: CD3, CD20	Maturity	Human	(50)
	EC	IHC: CD23, CD20, CD4, CD8, CD38	Maturity	Human	(58)
	ESCC	IHC: CD20, CD21, CD23, CD8, CD3, PNAd	Maturity	Human	(69)
BCL6	Lung Adenocarcinoma	IF: CD3, CD4, CD20, CD21, Bcl6	Maturity	Human	(70)
BCL6 + AID	PDAC	Human: IF: CD20, CD3, PNAd, CD21, BCL6, B220, AID, CD4, CD8, TIA, DC-LAMP; IHC: CD20, CD4, CD8, TIA, DC-LAMP, CD21, BCL6 Mice: IF: CD3, CD21, B220	Presence	Human and mice	(60)
Ki67	NSCC	IHC: DC-LAMP, CD8, Ki67, CD20	Density	Human	(71)
CD23 + BCL6 + PNAd	RCC	IF: CD23, CD20, BCL6, PNAd	Presence	Human	(31)
DC-LAMP	lung adenocarcinoma	IHC: CD3, CD20, DC-LAMP	Maturity	Human	(61)

AID, cytidine deaminase; FOXP3, forkhead box P3; BCL, B-cell lymphoma 6 protein; HE, hematoxylin and staining; IHC, immunohistochemistry; IF, immunofluorescence; DC, dendritic cells; PDAC, pancreatic ductal adenocarcinoma; OTSCC, oral tongue squamous cell carcinoma; LSCC, Lung Squamous Cell Carcinoma; ESCC, oesophageal squamous cell carcinoma; CCA, cholangiocarcinoma; CMM, Cutaneous melanoma metastases; UC, urothelial cancer; EBVaGC, Epstein-Barr virus-associated gastric cancer; EC, endometrial cancer; OSCC, oesophageal squamous cell carcinoma; PC, Pancreatic Cancer; NSCC, non-small cell lung cancer; RCC, renal cell cancer; HGSC, high-grade serous ovarian cancer; cSCC, cutaneous squamous cell carcinoma.

Furthermore, the maturity of TLS can be determined through genomic or transcriptomic data analysis. A 29-gene TLS signature, enriched with immunoglobulin genes and plasma cell-related genes that reflect the B cell differentiation into plasma cells, has been utilized to identify the presence of mature TLS (31). Ahn et al. observed elevated levels of 13 proteins (CYRIA, ENG, GPI, HLA-A, LIMA1, LRBA, LST1, MCAM, MGLL, NID1, NME2, PIK3R1, and STARD7) in mature TLS compared to immature TLS in hepatocellular carcinoma, suggesting their potential as markers of TLS maturity (72). Future research should focus on exploring gene or transcriptomic data that can accurately assess the maturity of TLS. This avenue of investigation holds promise for enhancing our understanding of TLS development and function.

## 6 Induction of TLS

**Figure 3:**
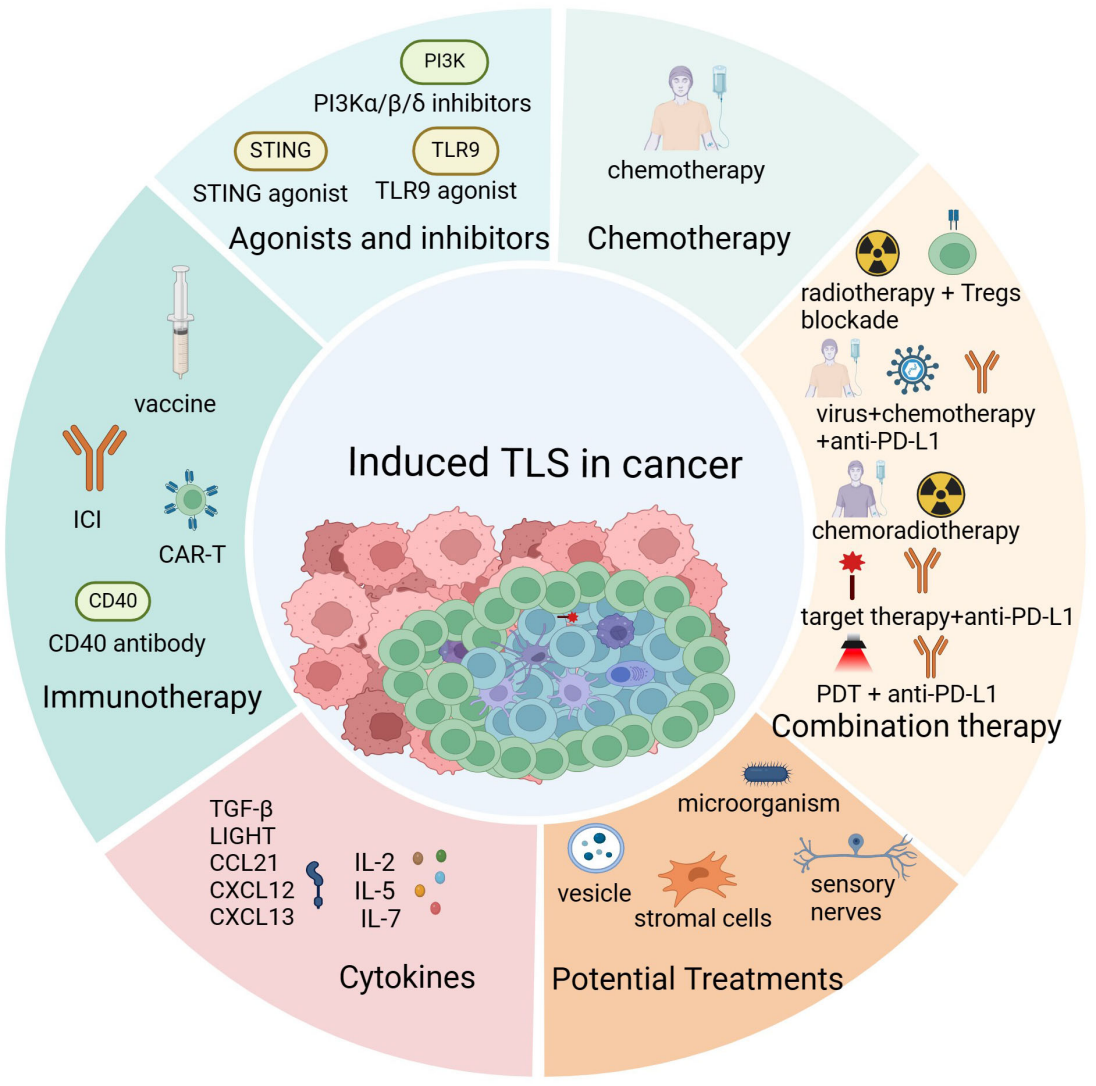
The induction of tertiary lymphoid structures. Cancer treatments promote the formation of tertiary lymphoid structures (TLS) mainly including immunotherapy, chemoradiotherapy, injection of TLS-associated cytokines and chemokines, activation of TLS-associated proteins and signaling pathways and some potential therapeutic approaches such as microbial transplantation and exogenous vesicle injection. Created with BioRender.com.

TLS has been found to be induced by different cancer treatments, and it was associated with prognosis and drug efficacy in most solid cancers. Consequently, it is crucial to understand the effect of treatment on TLS formation and function. Here, we compiled a summary of treatment methods capable of inducing TLS formation as follows (**Figure 3**).

### 6.1 Immunotherapy

Immunotherapy, including cancer vaccine, immune checkpoint inhibitors (ICI), oncolytic virotherapy, and chimeric antigen receptor T cell therapy (CAR-T), aims to enhance the immune response for improved antitumor immunity. TLS, an aggregate of immune cells in the TME, has been found to be induced after immunotherapy, especially after the vaccine. Maldonado et al. found that after intramuscular injection of a therapeutic vaccine targeted against HPV16E6/E7 antigen, a large number of T and B cells accumulated in the cervical interstitium of patients with cervical cancer, resulting induction of immune cell aggregates known as TLS (14). Similarly, intratumoral TLS was formed in 85% of patients with pancreatic cancer after administering an irradiated allogeneic vaccine secreted by granulocyte-macrophage colony-stimulating factor for pancreatic ductal adenocarcinoma (PDAC) and its presence improved the immunosuppressive state characteristic of PDAC (15). Incomplete Freund’s adjuvant, a commonly used adjuvant, was employed to amplify the immune response elicited by vaccines (73). Repeated administration of peptide vaccines formulated with incomplete Freund’s adjuvant could enhance the Th1-dominated immune response and improve immunogenicity. This effect has been associated with increased CD40L expression on CD4^+^ T cells, accumulation of mature DC, and upregulation of TLR adaptor protein expression. More importantly, it was reported to triggered TLS formation in melanoma patients (74).

Some functional research has supported the phenomenon of the induction of TLS by immunotherapy in clinical practice. In a glioma mouse model, TLS can be triggered near the meninges by systemic delivery of αCD40 in a CD11b^+^ B cell-dependent manner, which is expected to improve the immunosuppressive TME of glioma (75). In B16-OVA mice, treatment with either anti-PD-L1 monotherapy or combined anti-cytotoxic T lymphocyte antigen 4(CTLA-4) and anti-PD-1 therapy resulted in a substantial increase in the abundance and dimensions of TLS. These treatments were found to increase the number of T cells in TLS, potentially inducing the alterations of the cellular architecture of TLS. Notably, TLS had a more apparent cellular zone and GC with bright and dark zones after receiving ICI treatment, representing a more mature stage of TLS being induced (76, 77). The introduction of new materials can better assist vaccines to induce the formation of TLS. An immunomodulator-loaded porous 3D scaffold vaccine was able to better recruit numerous immune cells to form TLS and enhanced anti-tumor effects of ICI in melanoma mice (78).

In conclusion, researchers have observed that TLS can be formed in response to immunotherapy, but the specific mechanisms need to be further studied.

### 6.2 Cytokines

Exogenous administration and endogenous production of TLS-related cytokines have been reported to efficiently induce the formation of TLS, the most common of which are LIGHT and CXCL13. LIGHT, a TNF member expressed on activated T cells, interacts with HVEM and LTβR receptors. Binding to HVEM provides a co-stimulatory signal to T cells, while binding to LTβR promotes cytokine secretion and immune cell recruitment, contributing to TLS formation (79). LIGHT-vascular targeting peptide, delivering mouse LIGHT protein to tumor blood vessels, induced TLS formation, normalized tumor vascularity, suppressed tumor growth, and prolonged mouse survival. Addition of the Tag-Cpg-ODN vaccine enhances anti-tumor therapy efficacy (80). In PDAC mice, an anti-fibroblastic protein nanoparticle encoding LIGHT inhibited abnormal collagen secretion by fibroblasts, facilitating cytotoxic T lymphocyte recruitment, normalizing tumor vasculature, stimulating chemokine expression, and inducing intratumoral TLS (81). Transfection of LIGHT-overexpressing lentivirus into 4MOSC1 cells, followed by sublingual injection into HPV-head and neck squamous cell carcinoma mice, induced TLS formation and reduced tumor progression compared to the empty vector transfection group (82). Systemic administration of LIGHT through brain endothelial cell-targeting adeno-associated viral vectors induced HEV and T cell-rich TLS, decreased T cell exhaustion, and enhanced the presence of stem-like T cells in TLS. It also extended the survival of mice with PD-1 blocking-resistant glioma (83). Co-expression of LIGHT CAR-T cells enhanced efficacy by promoting immune cell aggregation and TLS formation. Transfer of humanized LIGHT-overexpressing OT-1 T cells into B16F10-OVA mice results in increased infiltration of T cells, DC, and B cells in tumors, the formation of highly vascular endothelial venules, significant tumor inhibition, and prolonged survival (84).

CXCL13 plays a crucial role in the initiation and maturation of TLS. Its secretion is influenced by the maturation stage of TLS. RNA ISH staining of high-grade serous ovarian cancer revealed that CXCL13 was predominantly secreted by CD4^+^ T cells in immature state, and FDC in mature state (55). A spongiform collagen scaffold with slow-release gel beads containing LTα1β2, CCL19, CCL21, CXCL12, CXCL13, and soluble RANK ligand promoted TLS formation and induced antigen-specific secondary immune responses (85). Transplantation of these induced TLS resulted in a robust and persistent antigen-specific humoral response, particularly in severe combined immunodeficiency mice, with functional B cells present (86). Concurrent intratumoral injection of CXCL13 and CCL21 increased the influx of CC-chemokine receptor 5 (CCR5)-expressing B and CCR5/CCR7-expressingT cells in the TME, inducing TLS formation and resulting in reduced tumor progression in PDAC mice (60). Additionally, intraperitoneal injection of recombinant CXCL13 induced TLS formation and extended survival time compared with PBS-treated ovarian cancer mice (55). Intraperitoneal injection of CXCL13 also enhanced the anti-tumor effect of anti-PD-1 treatment depending on the infiltration of CXCR5^+^ CD8^+^ T cells in high-grade serous ovarian cancer mice (87).

Furthermore, other cytokines have been reported to induce TLS formation. IL-7 plays a crucial role in the formation and maturation of TLS and can be used as an adjuvant to enhance the efficacy of vaccines. IL-7 adjuvanted vaccine caused overexpression of chemokines, enrich DC, macrophages, natural killer cells, B cells, and T cells in the lamina propria of the monkey vagina, and further induced the formation of TLS (88). Transforming Growth Factor-β downregulated the expression of Satb1 on CD4^+^ T cells, promoting Tfh cell formation and derepressing ICOS expression and T follicular regulatory cell development. Additionally, the cytokine-rich tumor microenvironment generated by Tfh cells was adequate to induce TLS formation in ovarian tumors in situ (89). IL-15, delivered through an oncolytic adenovirus, stimulated the proliferation and infiltration of DC, T cells, and natural killer cells in the TME. This cytokine activation also facilitated the normalization of tumor vasculature and the formation of TLS by activating the STING-TBK1-IRF3 pathway in DC (90). Non-replicating adenoviruses encoding mTNFa and mIL-2 could initiate gene signatures associated with TLS and GC formation, and significantly enhanced the humoral response in anti-PD-L1/anti-PD-1 refractory head and neck cancer (91). Intratumoral injection of IL-6-secreting DC has been shown to induce TLS production and effectively inhibit tumor progression in melanoma mice (92).

### 6.3 Agonists and inhibitors

The activation or inhibition of immune-related signaling pathways may cause immune cell aggregation and TLS formation, contributing positively to tumor immune surveillance and clearance. Intermittent administration of PI3Kα/β/δ inhibitors (BAY1082439) induced the clonal proliferation and anti-inflammatory phenotype in CD8^+^ T cells, and the formation of intratumoral TLS. The sustained anti-tumor effect of CD8^+^ T cells after drug withdrawal was likely related to the formation of TLS, as indicated by a large number of BrdU^+^ CD8^+^ T cells were present in TLS and a positive correlation between TLS score and CD8A expression in BAY1082439-treated TEN-null prostate cancer (93). Toll-like Receptor 9 (TLR9) is a receptor known for its ability to trigger the innate immune response. TLR9 agonists can activate plasmacytoid dendritic cells, leading to the recruitment of a large number of CD8^+^ T cells, DC, and the production of nearby antibodies through the generation of substantial amounts of IFN-α and other cytokines (94). Stimulator of interferon genes (STING), a pivotal signal transduction molecule in the innate immune response, can be activated by cytoplasmic DNA from pathogens and hosts. This activation leads to the secretion of IFN-1 and pro-inflammatory cytokines, thereby bolstering the anti-tumor immune response. Intratumoral injection of ADU S-100, a STING agonist, promoted tumor vascular normalization and TLS formation in B16F10 melanoma mice. This effect was connected with the upregulation of TLS-induced cytokines (CCL19, CCL21, LTα, LTβ, and LIGHT) and DC maturation facilitated by ADU S-100 (95). STING and TLR9 pathways-activating hydrogel loaded with CpG, Zn+, and CSNPs promoted the secretion of CXCL13, CCL19 and CCL21, initiating the development of HEV and the formation of TLS. It caused the increased of CD4^+^ T cells, CD8^+^ T cells, CD20^+^ B cells and M1-type macrophages and the decrease of Tregs, M2-type macrophages and myeloid-derived suppressor cells in the TME of melanoma mice (96). The nano-vaccine, consisting of Epstein-Barr virus nuclear antigen 1, CpG, and Mn2+, activated the STING and TLR9 pathways. It potently activated T cells, B cells and DC, and induced HEV formation. Furthermore, the nano-vaccine promoted the secretion of CCL19, CCL21 and CXCL13 in the TME, triggering intratumoral TLS formation, and markedly prolonging the survival time of nasopharyngeal carcinoma mice (97). Therefore, the activation of STING and TLR9 pathways can induce HEV development and TLS formation, remodel the TME, and enhance antitumor immune responses.

### 6.4 Chemotherapy

Chemotherapy employs chemical drugs to eradicate tumor cells and impede their growth and proliferation. The anti-tumor effects of chemotherapy encompass interference with DNA synthesis and cell division, induction of apoptosis, and enhancement of the immune system (98). Chemotherapeutic agents are categorized into immunogenic cell death (ICD) and non-ICD types based on their ability to initiate immune responses (99). Currently, chemotherapeutic drugs known to induce TLS primarily belong to the ICD category, such as 5-Fluorouracil, Oxaliplatin, Platinum-based drugs, and Doxorubicin. A small clinical study investigating hepatoblastoma in patients with adenomatous polyposis coli germline mutation reported the creation of extensive intratumoral TLS following cisplatin administration, whereas these lymphoid structures were absent in the pre-chemotherapy samples (11/11 vs. 0/5) (100). Chemotherapy has been observed to impact TLS components. In PDAC patients treated with ICD-chemotherapy, higher proportions of CD8^+^ T cells, PNAd^+^ HEV, macrophages, and Ki-67^+^ cells were observed within TLS. Additionally, these patients exhibited lower PD-1^+^ immunosuppressive lymphocytes and a more favorable prognosis compared to the surgical group (101). In melanoma mice, subcutaneous doxorubicin treatment increased CD8^+^ T cell infiltration, enhanced secretion of IFN-γ, granzyme B, and perforin, activated B cells and DC, promoted HEV, and induced TLS formation, surpassing the effects of non-ICD drugs and combination therapy (102). Moreover, the synergistic application of radiotherapy and chemotherapy exerted an enhanced impact on the immune response, as mentioned in subsequent combination therapy. This approach potentially facilitates the formation of TLS and modifies its characteristics. In summary, chemotherapy can modify the TME, triggering TLS formation, and augmenting its antitumor efficacy, primarily through the induction of immunogenic cell death.

### 6.5 Combination therapy

Preoperative chemoradiotherapy (NACRT) was thought to impact the cellular composition of the TME and recent evidence suggests that it also affects the immune cell composition within TLS (103–105). The proportion of CD8^+^ T cells, PNAd^+^ HEV, macrophages, and Ki67^+^ cells within TLS was significantly increased in the NACRT group compared to the surgical group. Additionally, the NACRT group had a longer OS in patients with PDAC when compared to the surgical group (101). In a phase 2 clinical trial, 15 TLS-negative soft tissue sarcoma patients received a combined treatment involving oncolytic vaccinia (JX-594), cyclophosphamide, and anti-PD-L1. The results revealed a 3.9-fold increase in the percentage of CD8^+^ T cells in the TME and upregulation of proteins related to T cell immune response and toxicity in plasma samples compared with baseline. These findings suggest that the combination therapy may ameliorate the immunosuppressive TME in soft tissue sarcomas. However, its potential to induce TLS formation remains unclear and warrants further investigation (106). Some evidence supporting TLS induction by the combination therapy has been observed in preclinical models. Glioma poses a significant challenge due to its immunosuppressive microenvironment. Studies have shown that combining radiotherapy with targeted Tregs interventions induces massive T cell-infiltration and the development of meningeal TLS, effectively activating the TME in preclinical glioma models (16). Low-dose radiotherapy has been found to trigger the development of immature TLS in a mouse model of lung cancer. Subsequent combined treatment involving low-dose radiotherapy and anti-PD-1 has shown a substantial enhancement in the maturation of TLS within mouse lung adenocarcinoma, leading to a more potent anti-tumor response. This effect is closely associated with the increased presence of CD8^+^ T cells within the TLS. Preclinical experiments and single-cell RNA sequencing (scRNA-seq) analysis of post-treatment thyroid tumor patients revealed that the combination of anti-PD-1 treatment and famitinib, which targets VEGF/PDGF signaling, effectively induces TLS in thyroid tumors (107).

Photodynamic therapy (PDT) is a technique that combines a photosensitizer and an appropriate light source to selectively destroy diseased tissue by inducing a photodynamic response dependent on the presence of oxygen. The advantages of PDT, including minimal invasiveness, repeatability, and targeted specificity towards pathological tissue, render it a highly promising therapeutic approach. Through the utilization of the Mouse Transcriptome Assay 1.0 chip, followed by validation using Western blotting, it was revealed that the therapeutic mechanism of PDT in cutaneous squamous cell carcinoma (cSCC) primarily involves immune regulation, as well as the NF-κB, TLR, PI3K-Akt, TNF, and MAPK signaling pathways (108). Aminolevulinic acid-based PDT (ALA-PDT) enhanced the anti-tumor effect of PD-L1 blockade in a mouse model of transplanted cSCC. The combination of ALA-PDT and anti-PD-L1 induced an increase in the chemokines CCL2, CCL8, CCL19, CCL21a, CCL21b, CXCL9 and CXCL13, and increased the density and maturity of TLS (109).

### 6.6 Other potential treatments

Other stimuli can also induce TLS formation, such as stromal cells transplantion, intestinal microorganism transplantation, cutaneous sensory nerve removal, and injection of exosome-derived vesicles. These methods are expected to open up new avenues for cancer treatment. In 2004, Watanabe’s group successfully induced the formation of GC-containing TLS by transplanting a spongy bovine collagen scaffold containing stromal cells into the subcapsular space of a mouse kidney (110). Upon subcutaneous injection of stromal cells derived from lymph nodes and expressing fibroblast markers into the dorsal region of mice, TLS formation was observed within 1.5 months (111). Transplantation of Helicobacter hepaticus (Hhep) into colorectal cancer mice increased infiltration of anti-tumor immune cells, especially Hhep-specific Tfh cells. This inhibited tumor progression through a Hhep-specific Tfh cell-dependent immune response. Hhep-colonized colorectal cancer mice showed elevated expression of genes related to TLS formation, leading to increased and mature intratumoral and peritumoral TLS, along with distinct T and B cell compartments within TLS (112). A recent study found that removing sensory nerves in the skin inhibited melanoma growth in mice by enhancing anti-tumor immune responses. This depletion led to increased T cell activation, recruitment of immune cells, and upregulation of pro-TLS cytokines (CCL3, CCL5, CCL19, CCL21, CXCL9, CXCL13, and TNFα) and LTβR agonists (LTα, LTβ, and LIGHT), promoting the formation of HEV and TLS (113). Additionally, intravenous injection of exosome-derived vesicles from vascular injuries induced TLS formation in aortic-allograft mice, demonstrating the critical role of γδT17 cells in TLS formation through reduced TLS formation in γδT17-deficient mice (114). Benzo(a)pyrene, the major component of tobacco, promoted the transcription of CCL21 in an aryl hydrocarbon receptor-dependent manner, leading to increased CD11a expression in CD4^+^ T cells via the CCL21/CCR7 axis. This, in turn, promoted interactions with CD20^+^ B cells and facilitated GC formation of TLS (115).

## 7 Challenges and perspectives

The induction and formation of TLS in tumors is a dynamic process influenced by various factors. Certain tumor therapies have been observed to trigger TLS formation, and their presence has been linked to treatment efficacy, suggesting that TLS could serve as a predictive biomarker for cancer treatment. Although the significance of TLS in cancer is now widely recognized, there are still many mysteries to be lifted. Firstly, further research is required to confirm and optimize the maturity criteria of TLS, which will serve as a crucial evaluation parameter in future clinical studies. Secondly, investigations into potential differences between pre-existing tumor TLS and TLS induced after treatment, as well as whether these mechanisms are identical, are warranted. Thirdly, while methods to provoke TLS formation have been reported, further research is imperative to enhance the maturity of these structures and optimize their functionality. Finally, progress in mechanistic TLS studies has been impeded by the absence of reliable *in vitro* systems and animal models. Discrepancies between mouse TLS models and clinical models, such as atypical GC formation in mouse TLS (60), necessitate further exploration to determine the applicability of existing mouse TLS models in clinical research. One promising avenue for addressing these challenges in the future may involve the use of organoids with immune properties. Our review summarizes previous criteria and markers for TLS maturity and classification, and further focus on the treatments that can induce TLS formation, laying the foundation for future research on the mechanism of TLS and its potential for personalized treatment.

## Author contributions

YC: Conceptualization, Visualization, Writing – original draft, Writing – review & editing. YW: Investigation, Methodology, Writing – review & editing. GY: Formal analysis, Software, Writing – review & editing. GZ: Funding acquisition, Project administration, Resources, Supervision, Writing – review & editing.

## References

[1] Gago da Graça C van Baarsen LGM Mebius RE. Tertiary lymphoid structures: diversity in their development, composition, and role. J Immunol. (2021) 206:273–81. doi: 10.4049/jimmunol.2000873 33397741

[2] Barone F Gardner DH Nayar S Steinthal N Buckley CD Luther SA. Stromal fibroblasts in tertiary lymphoid structures: A novel target in chronic inflammation. Front Immunol. (2016) 7:477. doi: 10.3389/fimmu.2016.00477 27877173 PMC5100680

[3] Kroeger DR Milne K Nelson BH. Tumor-infiltrating plasma cells are associated with tertiary lymphoid structures, cytolytic T-cell responses, and superior prognosis in ovarian cancer. Clin Cancer Res. (2016) 22:3005–15. doi: 10.1158/1078-0432.Ccr-15-2762 26763251

[4] Schumacher TN Thommen DS. Tertiary lymphoid structures in cancer. Science. (2022) 375:eabf9419. doi: 10.1126/science.abf9419 34990248

[5] Denton AE Carr EJ Magiera LP Watts AJB Fearon DT. Embryonic fap(+) lymphoid tissue organizer cells generate the reticular network of adult lymph nodes. J Exp Med. (2019) 216:2242–52. doi: 10.1084/jem.20181705 PMC6780995 31324739

[6] Sato Y Silina K van den Broek M Hirahara K Yanagita M. The roles of tertiary lymphoid structures in chronic diseases. Nat Rev Nephrol. (2023) 19:525–37. doi: 10.1038/s41581-023-00706-z PMC10092939 37046081

[7] Arroz-Madeira S Bekkhus T Ulvmar MH Petrova TV. Lessons of vascular specialization from secondary lymphoid organ lymphatic endothelial cells. Circ Res. (2023) 132:1203–25. doi: 10.1161/circresaha.123.322136 PMC10144364 37104555

[8] Cabrita R Lauss M Sanna A Donia M Skaarup Larsen M Mitra S. Tertiary lymphoid structures improve immunotherapy and survival in melanoma. Nature. (2020) 577:561–5. doi: 10.1038/s41586-019-1914-8 31942071

[9] Sarti Kinker G da Silva Medina T. Tertiary lymphoid structures as hubs of antitumour immunity. Nat Rev Cancer. (2023) 23:803. doi: 10.1038/s41568-023-00626-x 37758859

[10] Meylan M Petitprez F Lacroix L Di Tommaso L Roncalli M Bougoüin A. Early hepatic lesions display immature tertiary lymphoid structures and show elevated expression of immune inhibitory and immunosuppressive molecules. Clin Cancer Res. (2020) 26:4381–9. doi: 10.1158/1078-0432.Ccr-19-2929 32269054

[11] Tietscher S Wagner J Anzeneder T Langwieder C Rees M Sobottka B. A comprehensive single-cell map of T cell exhaustion-associated immune environments in human breast cancer. Nat Commun. (2023) 14:98. doi: 10.1038/s41467-022-35238-w 36609566 PMC9822999

[12] Sautès-Fridman C Petitprez F Calderaro J Fridman WH. Tertiary lymphoid structures in the era of cancer immunotherapy. Nat Rev Cancer. (2019) 19:307–25. doi: 10.1038/s41568-019-0144-6 31092904

[13] Helmink BA Reddy SM Gao J Zhang S Basar R Thakur R. B cells and tertiary lymphoid structures promote immunotherapy response. Nature. (2020) 577:549–55. doi: 10.1038/s41586-019-1922-8 PMC8762581 31942075

[14] Maldonado L Teague JE Morrow MP Jotova I Wu TC Wang C. Intramuscular therapeutic vaccination targeting hpv16 induces T cell responses that localize in mucosal lesions. Sci Trans Med. (2014) 6. doi: 10.1126/scitranslmed.3007323 PMC4086631 24477000

[15] Lutz ER Wu AA Bigelow E Sharma R Mo G Soares K. Immunotherapy converts nonimmunogenic pancreatic tumors into immunogenic foci of immune regulation. Cancer Immunol Res. (2014) 2:616–31. doi: 10.1158/2326-6066.Cir-14-0027 PMC4082460 24942756

[16] van Hooren L Handgraaf SM Kloosterman DJ Karimi E van Mil L Gassama AA. Cd103(+) regulatory T cells underlie resistance to radio-immunotherapy and impair Cd8(+) T cell activation in glioblastoma. Nat Cancer. (2023) 4:665–81. doi: 10.1038/s43018-023-00547-6 PMC10212765 37081259

[17] Li H Ding JY Zhang MJ Yu HJ Sun ZJ. Tertiary lymphoid structures and cytokines interconnections: the implication in cancer immunotherapy. Cancer Lett. (2023) 568:216293. doi: 10.1016/j.canlet.2023.216293 37392991

[18] Deteix C Attuil-Audenis V Duthey A Patey N McGregor B Dubois V. Intragraft Th17 infiltrate promotes lymphoid neogenesis and hastens clinical chronic rejection. J Immunol. (2010) 184:5344–51. doi: 10.4049/jimmunol.0902999 20357253

[19] Nayar S Campos J Chung MM Navarro-Núñez L Chachlani M Steinthal N. Bimodal expansion of the lymphatic vessels is regulated by the sequential expression of Il-7 and lymphotoxin A1β2 in newly formed tertiary lymphoid structures. J Immunol. (2016) 197:1957–67. doi: 10.4049/jimmunol.1500686 PMC4991245 27474071

[20] Wang ZZ Song J Wang H Li JX Xiao Q Yu Z. Stromal cells and B cells orchestrate ectopic lymphoid tissue formation in nasal polyps. Allergy. (2021) 76:1416–31. doi: 10.1111/all.14612 33022771

[21] Hua Y Vella G Rambow F Allen E Antoranz Martinez A Duhamel M. Cancer Immunotherapies Transition Endothelial Cells into Hevs That Generate Tcf1(+) T Lymphocyte Niches through a Feed-Forward Loop. Cancer Cell. (2022) 40:1600–18.e10. doi: 10.1016/j.ccell.2022.11.002 36423635 PMC9899876

[22] Yoshimoto K Tanaka M Kojima M Setoyama Y Kameda H Suzuki K. Regulatory mechanisms for the production of Baff and Il-6 are impaired in monocytes of patients of primary Sjögren's syndrome. Arthritis Res Ther. (2011) 13:R170. doi: 10.1186/ar3493 22018243 PMC3308105

[23] Denton AE Innocentin S Carr EJ Bradford BM Lafouresse F Mabbott NA. Type I interferon induces Cxcl13 to support ectopic germinal center formation. J Exp Med. (2019) 216:621–37. doi: 10.1084/jem.20181216 PMC6400543 30723095

[24] Sato Y Oguchi A Fukushima Y Masuda K Toriu N Taniguchi K. Cd153/Cd30 signaling promotes age-dependent tertiary lymphoid tissue expansion and kidney injury. J Clin Invest. (2022) 132:e146071. doi: 10.1172/jci146071 34813503 PMC8759786

[25] Fridman WH Meylan M Pupier G Calvez A Hernandez I Sautès-Fridman C. Tertiary lymphoid structures and B cells: an intratumoral immunity cycle. Immunity. (2023) 56:2254–69. doi: 10.1016/j.immuni.2023.08.009 37699391

[26] Buisseret L Garaud S de Wind A Van den Eynden G Boisson A Solinas C. Tumor-infiltrating lymphocyte composition, organization and Pd-1/ Pd-L1 expression are linked in breast cancer. Oncoimmunology. (2017) 6:e1257452. doi: 10.1080/2162402x.2016.1257452 28197375 PMC5283629

[27] Yang M Che Y Li K Fang Z Li S Wang M. Detection and quantitative analysis of tumor-associated tertiary lymphoid structures. J Zhejiang Univ Sci B. (2023) 24:779–95. doi: 10.1631/jzus.B2200605 PMC10500099 37701955

[28] Coppola D Nebozhyn M Khalil F Dai H Yeatman T Loboda A. Unique ectopic lymph node-like structures present in human primary colorectal carcinoma are identified by immune gene array profiling. Am J Pathol. (2011) 179:37–45. doi: 10.1016/j.ajpath.2011.03.007 21703392 PMC3123872

[29] Becht E Giraldo NA Lacroix L Buttard B Elarouci N Petitprez F. Estimating the population abundance of tissue-infiltrating immune and stromal cell populations using gene expression. Genome Biol. (2016) 17:218. doi: 10.1186/s13059-016-1070-5 27765066 PMC5073889

[30] Petitprez F de Reyniès A Keung EZ Chen TW Sun CM Calderaro J. B cells are associated with survival and immunotherapy response in sarcoma. Nature. (2020) 577:556–60. doi: 10.1038/s41586-019-1906-8 31942077

[31] Meylan M Petitprez F Becht E Bougoüin A Pupier G Calvez A. Tertiary lymphoid structures generate and propagate anti-tumor antibody-producing plasma cells in renal cell cancer. Immunity. (2022) 55:527–41.e5. doi: 10.1016/j.immuni.2022.02.001 35231421

[32] Gu-Trantien C Loi S Garaud S Equeter C Libin M de Wind A. Cd4^+^ Follicular helper T cell infiltration predicts breast cancer survival. J Clin Invest. (2013) 123:2873–92. doi: 10.1172/jci67428 PMC3696556 23778140

[33] Hennequin A Derangère V Boidot R Apetoh L Vincent J Orry D. Tumor infiltration by Tbet+ Effector T cells and Cd20+ B cells is associated with survival in gastric cancer patients. Oncoimmunology. (2016) 5:e1054598. doi: 10.1080/2162402x.2015.1054598 27057426 PMC4801425

[34] Ma Y Li X Zhang J Zhao X Lu Y Shen G. Integrating tertiary lymphoid structure-associated genes into computational models to evaluate prognostication and immune infiltration in pancreatic cancer. J Leukoc Biol. (2024) Online ahead of print. doi: 10.1093/jleuko/qiae067 38484172

[35] Bressan D Battistoni G Hannon GJ. The dawn of spatial omics. Science. (2023) 381:eabq4964. doi: 10.1126/science.abq4964 37535749 PMC7614974

[36] Li Z Jiang Y Li B Han Z Shen J Xia Y. Development and validation of a machine learning model for detection and classification of tertiary lymphoid structures in gastrointestinal cancers. JAMA Netw Open. (2023) 6:e2252553. doi: 10.1001/jamanetworkopen.2022.52553 36692877 PMC10408275

[37] Wang Y Lin H Yao N Chen X Qiu B Cui Y. Computerized tertiary lymphoid structures density on H&E-images is a prognostic biomarker in resectable lung adenocarcinoma. iScience. (2023) 26:107635. doi: 10.1016/j.isci.2023.107635 37664636 PMC10474456

[38] Desbois M Udyavar AR Ryner L Kozlowski C Guan Y Dürrbaum M. Integrated digital pathology and transcriptome analysis identifies molecular mediators of T-cell exclusion in ovarian cancer. Nat Commun. (2020) 11:5583. doi: 10.1038/s41467-020-19408-2 33149148 PMC7642433

[39] Joshi NS Akama-Garren EH Lu Y Lee DY Chang GP Li A. Regulatory T cells in tumor-associated tertiary lymphoid structures suppress anti-tumor T cell responses. Immunity. (2015) 43:579–90. doi: 10.1016/j.immuni.2015.08.006 PMC4826619 26341400

[40] Houel A Foloppe J Dieu-Nosjean MC. Harnessing the power of oncolytic virotherapy and tertiary lymphoid structures to amplify antitumor immune responses in cancer patients. Semin Immunol. (2023) 69:101796. doi: 10.1016/j.smim.2023.101796 37356421

[41] Devi-Marulkar P Fastenackels S Karapentiantz P Goc J Germain C Kaplon H. Regulatory T cells infiltrate the tumor-induced tertiary lymphoïd structures and are associated with poor clinical outcome in Nsclc. Commun Biol. (2022) 5:1416. doi: 10.1038/s42003-022-04356-y 36566320 PMC9789959

[42] Brunet M Crombé A Cousin S Vanhersecke L Le Loarer F Bessede A. Mature tertiary lymphoid structure is a specific biomarker of cancer immunotherapy and does not predict outcome to chemotherapy in non-small-cell lung cancer. Ann Oncol. (2022) 33:1084–5. doi: 10.1016/j.annonc.2022.06.007 35764273

[43] Wang Q Shen X An R Bai J Dong J Cai H. Peritumoral tertiary lymphoid structure and tumor stroma percentage predict the prognosis of patients with non-metastatic colorectal cancer. Front Immunol. (2022) 13:962056. doi: 10.3389/fimmu.2022.962056 36189233 PMC9524924

[44] Li H Liu H Fu H Li J Xu L Wang G. Peritumoral tertiary lymphoid structures correlate with protective immunity and improved prognosis in patients with hepatocellular carcinoma. Front Immunol. (2021) 12:648812. doi: 10.3389/fimmu.2021.648812 34122408 PMC8187907

[45] Sofopoulos M Fortis SP Vaxevanis CK Sotiriadou NN Arnogiannaki N Ardavanis A. The prognostic significance of peritumoral tertiary lymphoid structures in breast cancer. Cancer Immunol Immunother. (2019) 68:1733–45. doi: 10.1007/s00262-019-02407-8 PMC11028375 31598757

[46] Zhang T Lei X Jia W Li J Nie Y Mao Z. Peritumor tertiary lymphoid structures are associated with infiltrating neutrophils and inferior prognosis in hepatocellular carcinoma. Cancer Med. (2023) 12:3068–78. doi: 10.1002/cam4.5227 PMC9939159 36082777

[47] Ding GY Ma JQ Yun JP Chen X Ling Y Zhang S. Distribution and density of tertiary lymphoid structures predict clinical outcome in intrahepatic cholangiocarcinoma. J Hepatol. (2022) 76:608–18. doi: 10.1016/j.jhep.2021.10.030 34793865

[48] Shang T Jiang T Lu T Wang H Cui X Pan Y. Tertiary lymphoid structures predict the prognosis and immunotherapy response of cholangiocarcinoma. Front Immunol. (2023) 14:1166497. doi: 10.3389/fimmu.2023.1166497 37234171 PMC10206168

[49] Nie Y Fan H Li J Lei X Zhang T Wang Y. Tertiary lymphoid structures: associated multiple immune cells and analysis their formation in hepatocellular carcinoma. FASEB J. (2022) 36:e22586. doi: 10.1096/fj.202200269RR 36190431

[50] Vanhersecke L Brunet M Guégan JP Rey C Bougouin A Cousin S. Mature tertiary lymphoid structures predict immune checkpoint inhibitor efficacy in solid tumors independently of Pd-L1 expression. Nat Cancer. (2021) 2:794–802. doi: 10.1038/s43018-021-00232-6 35118423 PMC8809887

[51] Yin YX Ling YH Wei XL He CY Wang BZ Hu CF. Impact of mature tertiary lymphoid structures on prognosis and therapeutic response of Epstein-Barr virus-associated gastric cancer patients. Front Immunol. (2022) 13:973085. doi: 10.3389/fimmu.2022.973085 36591236 PMC9794571

[52] Werner F Wagner C Simon M Glatz K Mertz KD Läubli H. A standardized analysis of tertiary lymphoid structures in human melanoma: disease progression- and tumor site-associated changes with germinal center alteration. Front Immunol. (2021) 12:675146. doi: 10.3389/fimmu.2021.675146 34248957 PMC8264652

[53] van Dijk N Gil-Jimenez A Silina K van Montfoort ML Einerhand S Jonkman L. The tumor immune landscape and architecture of tertiary lymphoid structures in urothelial cancer. Front Immunol. (2021) 12:793964. doi: 10.3389/fimmu.2021.793964 34987518 PMC8721669

[54] Li R Huang X Yang W Wang J Liang Y Zhang T. Tertiary lymphoid structures favor outcome in resected esophageal squamous cell carcinoma. J Pathol Clin Res. (2022) 8:422–35. doi: 10.1002/cjp2.281 PMC9353661 35711130

[55] Ukita M Hamanishi J Yoshitomi H Yamanoi K Takamatsu S Ueda A. Cxcl13-producing Cd4+ T cells accumulate in the early phase of tertiary lymphoid structures in ovarian cancer. JCI Insight. (2022) 7:e157215. doi: 10.1172/jci.insight.157215 35552285 PMC9309049

[56] Wu YH Wu F Yan GR Zeng QY Jia N Zheng Z. Features and clinical significance of tertiary lymphoid structure in cutaneous squamous cell carcinoma. J Eur Acad Dermatol Venereol. (2022) 36:2043–50. doi: 10.1111/jdv.18464 35881141

[57] Posch F Silina K Leibl S Mündlein A Moch H Siebenhüner A. Maturation of tertiary lymphoid structures and recurrence of stage ii and iii colorectal cancer. Oncoimmunology. (2018) 7:e1378844. doi: 10.1080/2162402x.2017.1378844 29416939 PMC5798199

[58] Qin M Hamanishi J Ukita M Yamanoi K Takamatsu S Abiko K. Tertiary lymphoid structures are associated with favorable survival outcomes in patients with endometrial cancer. Cancer Immunol Immunother. (2022) 71:1431–42. doi: 10.1007/s00262-021-03093-1 PMC9123039 34689225

[59] Vanhersecke L Bougouin A Crombé A Brunet M Sofeu C Parrens M. Standardized pathology screening of mature tertiary lymphoid structures in cancers. Lab Invest. (2023) 103:100063. doi: 10.1016/j.labinv.2023.100063 36801637

[60] Delvecchio FR Fincham REA Spear S Clear A Roy-Luzarraga M Balkwill FR. Pancreatic cancer chemotherapy is potentiated by induction of tertiary lymphoid structures in mice. Cell Mol Gastroenterol Hepatol. (2021) 12:1543–65. doi: 10.1016/j.jcmgh.2021.06.023 PMC8529396 34252585

[61] Wakasu S Tagawa T Haratake N Kinoshita F Oku Y Ono Y. Preventive effect of tertiary lymphoid structures on lymph node metastasis of lung adenocarcinoma. Cancer Immunol Immunother. (2023) 72:1823–34. doi: 10.1007/s00262-022-03353-8 PMC10992259 36688996

[62] Ling Y Zhong J Weng Z Lin G Liu C Pan C. The prognostic value and molecular properties of tertiary lymphoid structures in oesophageal squamous cell carcinoma. Clin Transl Med. (2022) 12:e1074. doi: 10.1002/ctm2.1074 36245289 PMC9574489

[63] Silina K Soltermann A Attar FM Casanova R Uckeley ZM Thut H. Germinal centers determine the prognostic relevance of tertiary lymphoid structures and are impaired by corticosteroids in lung squamous cell carcinoma. Cancer Res. (2018) 78:1308–20. doi: 10.1158/0008-5472.CAN-17-1987 29279354

[64] Liang H Zhang Z Guan Z Zheng S Lou J Liu W. Follicle-like tertiary lymphoid structures: A potential biomarker for prognosis and immunotherapy response in patients with laryngeal squamous cell carcinoma. Front Immunol. (2023) 14:1096220. doi: 10.3389/fimmu.2023.1096220 36776859 PMC9912937

[65] Hayashi Y Makino T Sato E Ohshima K Nogi Y Kanemura T. Density and maturity of peritumoral tertiary lymphoid structures in oesophageal squamous cell carcinoma predicts patient survival and response to immune checkpoint inhibitors. Br J Cancer. (2023) 128:2175–85. doi: 10.1038/s41416-023-02235-9 PMC10241865 37016103

[66] Le Rochais M Hemon P Ben-Guigui D Garaud S Le Dantec C Pers JO. Deciphering the maturation of tertiary lymphoid structures in cancer and inflammatory diseases of the digestive tract using imaging mass cytometry. Front Immunol. (2023) 14:1147480. doi: 10.3389/fimmu.2023.1147480 37143660 PMC10151544

[67] Lynch KT Young SJ Meneveau MO Wages NA Engelhard VH Slingluff CL Jr.. Heterogeneity in tertiary lymphoid structure B-cells correlates with patient survival in metastatic melanoma. J Immunother Cancer. (2021) 9:e002273. doi: 10.1136/jitc-2020-002273 34103353 PMC8190052

[68] van Dijk N Gil-Jimenez A Silina K Hendricksen K Smit LA de Feijter JM. Preoperative ipilimumab plus nivolumab in locoregionally advanced urothelial cancer: the Nabucco trial. Nat Med. (2020) 26:1839–44. doi: 10.1038/s41591-020-1085-z 33046870

[69] Deguchi S Tanaka H Suzuki S Natsuki S Mori T Miki Y. Clinical relevance of tertiary lymphoid structures in esophageal squamous cell carcinoma. BMC Cancer. (2022) 22:699. doi: 10.1186/s12885-022-09777-w 35751038 PMC9233387

[70] Zhao H Wang H Zhao Y Sun Q Ren X. Tumor-resident T cells, associated with tertiary lymphoid structure maturity, improve survival in patients with stage iii lung adenocarcinoma. Front Immunol. (2022) 13:877689. doi: 10.3389/fimmu.2022.877689 35663939 PMC9161276

[71] Rakaee M Kilvaer TK Jamaly S Berg T Paulsen EE Berglund M. Tertiary lymphoid structure score: A promising approach to refine the Tnm staging in resected non-small cell lung cancer. Br J Cancer. (2021) 124:1680–9. doi: 10.1038/s41416-021-01307-y PMC8110789 33723388

[72] Ahn B Ahn HS Shin J Jun E Koh EY Ryu YM. Characterization of lymphocyte-rich hepatocellular carcinoma and the prognostic role of tertiary lymphoid structures. Liver Int. (2024). doi: 10.1111/liv.15865 38363048

[73] Melssen MM Fisher CT Slingluff CL Melief CJM. Peptide emulsions in incomplete Freund's adjuvant create effective nurseries promoting egress of systemic Cd4(+) and Cd8(+) T cells for immunotherapy of cancer. J Immunother Cancer. (2022) 10:e004709. doi: 10.1136/jitc-2022-004709 36939214 PMC9472143

[74] Pollack KE Meneveau MO Melssen MM Lynch KT Koeppel AF Young SJ. Incomplete Freund's adjuvant reduces arginase and enhances Th1 dominance, Tlr signaling and Cd40 ligand expression in the vaccine site microenvironment. J Immunother Cancer. (2020) 8:e000544. doi: 10.1136/jitc-2020-000544 32350119 PMC7213888

[75] van Hooren L Vaccaro A Ramachandran M Vazaios K Libard S van de Walle T. Agonistic Cd40 therapy induces tertiary lymphoid structures but impairs responses to checkpoint blockade in glioma. Nat Commun. (2021) 12:4127. doi: 10.1038/s41467-021-24347-7 34226552 PMC8257767

[76] Eschweiler S Clarke J Ramirez-Suastegui C Panwar B Madrigal A Chee SJ. Intratumoral follicular regulatory T cells curtail anti-Pd-1 treatment efficacy. Nat Immunol. (2021) 22:1052–63. doi: 10.1038/s41590-021-00958-6 PMC8434898 34168370

[77] Rodriguez AB Peske JD Woods AN Leick KM Mauldin IS Meneveau MO. Immune mechanisms orchestrate tertiary lymphoid structures in tumors *via* cancer-associated fibroblasts. Cell Rep. (2021) 36:109422. doi: 10.1016/j.celrep.2021.109422 34289373 PMC8362934

[78] Zhang Y Xu J Fei Z Dai H Fan Q Yang Q. 3d printing scaffold vaccine for antitumor immunity. Adv Mater. (2021) 33:e2106768. doi: 10.1002/adma.202106768 34601760

[79] Tang H Wang Y Chlewicki LK Zhang Y Guo J Liang W. Facilitating T cell infiltration in tumor microenvironment overcomes resistance to Pd-L1 blockade. Cancer Cell. (2016) 30:500. doi: 10.1016/j.ccell.2016.08.011 27622338

[80] Johansson-Percival A He B Li Z-J Kjellén A Russell K Li J. *De novo* induction of intratumoral lymphoid structures and vessel normalization enhances immunotherapy in resistant tumors. Nat Immunol. (2017) 18:1207–17. doi: 10.1038/ni.3836 28892469

[81] Huang Y Chen Y Zhou S Chen L Wang J Pei Y. Dual-mechanism based Ctls infiltration enhancement initiated by nano-sapper potentiates immunotherapy against immune-excluded tumors. Nat Commun. (2020) 11:622. doi: 10.1038/s41467-020-14425-7 32001695 PMC6992734

[82] Li H Zhu SW Zhou JJ Chen DR Liu J Wu ZZ. Tertiary lymphoid structure raises survival and immunotherapy in Hpv(-) Hnscc. J Dent Res. (2023) 102:678–88. doi: 10.1177/00220345231151685 36883630

[83] Ramachandran M Vaccaro A van de Walle T Georganaki M Lugano R Vemuri K. Tailoring vascular phenotype through Aav therapy promotes anti-tumor immunity in glioma. Cancer Cell. (2023) 41:1134–51.e10. doi: 10.1016/j.ccell.2023.04.010 37172581

[84] Zhang N Liu X Qin J Sun Y Xiong H Lin B. Light/Tnfsf14 promotes Car-T cell trafficking and cytotoxicity through reversing immunosuppressive tumor microenvironment. Mol Ther. (2023) 31:2575–90. doi: 10.1016/j.ymthe.2023.06.015 PMC10491984 37408308

[85] Kobayashi Y Watanabe T. Gel-trapped lymphorganogenic chemokines trigger artificial tertiary lymphoid organs and mount adaptive immune responses in vivo. Front Immunol. (2016) 7:316. doi: 10.3389/fimmu.2016.00316 27597851 PMC4992816

[86] Okamoto N Chihara R Shimizu C Nishimoto S Watanabe T. Artificial lymph nodes induce potent secondary immune responses in naive and immunodeficient mice. J Clin Invest. (2007) 117:997–1007. doi: 10.1172/jci30379 17364025 PMC1810575

[87] Yang M Lu J Zhang G Wang Y He M Xu Q. Cxcl13 shapes immunoactive tumor microenvironment and enhances the efficacy of Pd-1 checkpoint blockade in high-grade serous ovarian cancer. J Immunother Cancer. (2021) 9:e001136. doi: 10.1136/jitc-2020-001136 33452206 PMC7813306

[88] Groeneveld CS Fontugne J Cabel L Bernard-Pierrot I Radvanyi F Allory Y. Tertiary lymphoid structures marker Cxcl13 is associated with better survival for patients with advanced-stage bladder cancer treated with immunotherapy. Eur J Cancer (Oxford Engl 1990). (2021) 148:181–9. doi: 10.1016/j.ejca.2021.01.036 33743486

[89] Chaurio RA Anadon CM Lee Costich T Payne KK Biswas S Harro CM. Tgf-beta-mediated silencing of genomic organizer satb1 promotes Tfh cell differentiation and formation of intra-tumoral tertiary lymphoid structures. Immunity. (2022) 55:115–28.e9. doi: 10.1016/j.immuni.2021.12.007 35021053 PMC8852221

[90] He T Hao Z Lin M Xin Z Chen Y Ouyang W. Oncolytic adenovirus promotes vascular normalization and nonclassical tertiary lymphoid structure formation through sting-mediated dc activation. Oncoimmunology. (2022) 11:2093054. doi: 10.1080/2162402x.2022.2093054 35800155 PMC9255224

[91] Clubb JHA Kudling TV Heiniö C Basnet S Pakola S Cervera Carrascón V. Adenovirus encoding tumor necrosis factor alpha and interleukin 2 induces a tertiary lymphoid structure signature in immune checkpoint inhibitor refractory head and neck cancer. Front Immunol. (2022) 13:794251. doi: 10.3389/fimmu.2022.794251 35355980 PMC8959099

[92] Weinstein AM Chen L Brzana EA Patil PR Taylor JL Fabian KL. Tbet and Il-36γ Cooperate in therapeutic Dc-mediated promotion of ectopic lymphoid organogenesis in the tumor microenvironment. Oncoimmunology. (2017) 6:e1322238. doi: 10.1080/2162402x.2017.1322238 28680760 PMC5486180

[93] Qi Z Xu Z Zhang L Zou Y Li J Yan W. Overcoming resistance to immune checkpoint therapy in Pten-null prostate cancer by intermittent anti-Pi3kalpha/beta/delta treatment. Nat Commun. (2022) 13:182. doi: 10.1038/s41467-021-27833-0 35013322 PMC8748754

[94] Gallotta M Assi H Degagné É Kannan SK Coffman RL Guiducci C. Inhaled Tlr9 agonist renders lung tumors permissive to Pd-1 blockade by promoting optimal Cd4(+) and Cd8(+) T-cell interplay. Cancer Res. (2018) 78:4943–56. doi: 10.1158/0008-5472.Can-18-0729 29945961

[95] Chelvanambi M Fecek RJ Taylor JL Storkus WJ. Sting agonist-based treatment promotes vascular normalization and tertiary lymphoid structure formation in the therapeutic melanoma microenvironment. J Immunother Cancer. (2021) 9:e001906. doi: 10.1136/jitc-2020-001906 33526609 PMC7852948

[96] Jin XK Liang JL Zhang SM Ji P Huang QX Qin YT. Engineering metal-based hydrogel-mediated tertiary lymphoid structure formation via activation of the sting pathway for enhanced immunotherapy. Mater Horiz. (2023) 10:4365–79. doi: 10.1039/D3MH00748K 37455643

[97] Wen Z Liu H Qiao D Chen H Li L Yang Z. Nanovaccines fostering tertiary lymphoid structure to attack mimicry nasopharyngeal carcinoma. ACS Nano. (2023) 17:7194–206. doi: 10.1021/acsnano.2c09619 37057967

[98] Kelland L. The resurgence of platinum-based cancer chemotherapy. Nat Rev Cancer. (2007) 7:573–84. doi: 10.1038/nrc2167 17625587

[99] Li Z Lai X Fu S Ren L Cai H Zhang H. Immunogenic cell death activates the tumor immune microenvironment to boost the immunotherapy efficiency. Adv Sci (Weinh). (2022) 9:e2201734. doi: 10.1002/advs.202201734 35652198 PMC9353475

[100] Morcrette G Hirsch TZ Badour E Pilet J Caruso S Calderaro J. Apc germline hepatoblastomas demonstrate cisplatin-induced intratumor tertiary lymphoid structures. Oncoimmunology. (2019) 8:e1583547. doi: 10.1080/2162402X.2019.1583547 31069152 PMC6492969

[101] Kuwabara S Tsuchikawa T Nakamura T Hatanaka Y Hatanaka KC Sasaki K. Prognostic relevance of tertiary lymphoid organs following neoadjuvant chemoradiotherapy in pancreatic ductal adenocarcinoma. Cancer Sci. (2019) 110:1853–62. doi: 10.1111/cas.14023 PMC6549910 30997706

[102] Zhao H Zhao Y Zhang S Wang Z Yu W Dong N. Effects of immunogenic cell death-inducing chemotherapeutics on the immune cell activation and tertiary lymphoid structure formation in melanoma. Front Immunol. (2024) 15:1302751. doi: 10.3389/fimmu.2024.1302751 38384466 PMC10879401

[103] Tsuchikawa T Hirano S Tanaka E Matsumoto J Kato K Nakamura T. Novel aspects of preoperative chemoradiation therapy improving anti-tumor immunity in pancreatic cancer. Cancer Sci. (2013) 104:531–5. doi: 10.1111/cas.12119 PMC7657202 23363422

[104] Homma Y Taniguchi K Murakami T Nakagawa K Nakazawa M Matsuyama R. Immunological impact of neoadjuvant chemoradiotherapy in patients with borderline resectable pancreatic ductal adenocarcinoma. Ann Surg Oncol. (2014) 21:670–6. doi: 10.1245/s10434-013-3390-y 24310792

[105] Lu Y Zhao Q Liao JY Song E Xia Q Pan J. Complement signals determine opposite effects of B cells in chemotherapy-induced immunity. Cell. (2020) 180:1081–97.e24. doi: 10.1016/j.cell.2020.02.015 32142650

[106] Toulmonde M Guegan JP Spalato-Ceruso M Peyraud F Kind M Vanhersecke L. Reshaping the tumor microenvironment of cold soft-tissue sarcomas with oncolytic viral therapy: A phase 2 trial of intratumoral Jx-594 combined with avelumab and low-dose cyclophosphamide. Mol Cancer. (2024) 23:38. doi: 10.1186/s12943-024-01946-8 38378555 PMC10877825

[107] Han PZ Ye WD Yu PC Tan LC Shi X Chen XF. Distinct tumor microenvironment makes anaplastic thyroid cancer more lethal but immunotherapy-sensitive than papillary thyroid cancer. JCI Insight. (2024). doi: 10.1172/jci.insight.173712 PMC11141884 38478516

[108] Fang S Wu Y Zhang H Zeng Q Wang P Zhang L. Molecular characterization of gene expression changes in murine cutaneous squamous cell carcinoma after 5-aminolevulinic acid photodynamic therapy. Photodiagnosis Photodyn Ther. (2022) 39:102907. doi: 10.1016/j.pdpdt.2022.102907 35569747

[109] Zeng Q Yang J Ji J Wang P Zhang L Yan G. Pd-L1 blockade potentiates the antitumor effects of Ala-Pdt and optimizes the tumor microenvironment in cutaneous squamous cell carcinoma. Oncoimmunology. (2022) 11:2061396. doi: 10.1080/2162402x.2022.2061396 35402079 PMC8986186

[110] Suematsu S Watanabe T. Generation of a synthetic lymphoid tissue-like organoid in mice. Nat Biotechnol. (2004) 22:1539–45. doi: 10.1038/nbt1039 15568019

[111] Zhu G Nemoto S Mailloux AW Perez-Villarroel P Nakagawa R Falahat R. Induction of tertiary lymphoid structures with antitumor function by a lymph node-derived stromal cell line. Front Immunol. (2018) 9:1609. doi: 10.3389/fimmu.2018.01609 30061886 PMC6054958

[112] Overacre-Delgoffe AE Bumgarner HJ Cillo AR Burr AHP Tometich JT Bhattacharjee A. Microbiota-specific T follicular helper cells drive tertiary lymphoid structures and anti-tumor immunity against colorectal cancer. Immunity. (2021) 54:2812–24.e4. doi: 10.1016/j.immuni.2021.11.003 34861182 PMC8865366

[113] Vats K Kruglov O Sahoo B Soman V Zhang J Shurin GV. Sensory nerves impede the formation of tertiary lymphoid structures and development of protective antimelanoma immune responses. Cancer Immunol Res. (2022) 10:1141–54. doi: 10.1158/2326-6066.Cir-22-0110 PMC10314799 35834791

[114] Dieudé M Turgeon J Karakeussian Rimbaud A Beillevaire D Qi S Patey N. Extracellular vesicles derived from injured vascular tissue promote the formation of tertiary lymphoid structures in vascular allografts. Am J Transplant. (2020) 20:726–38. doi: 10.1111/ajt.15707 PMC7064890 31729155

[115] Yin X Wang H Li R Song X Zhang T Liang Y. Tobacco exposure primes the secretion of Ccl21 positively associated with tertiary lymphoid structure and response to immunotherapy. J Immunother Cancer. (2023) 11:e006939. doi: 10.1136/jitc-2023-006939 37369391 PMC10410842

